# Multidrug-resistant organisms (MDROs) in patients with subarachnoid hemorrhage (SAH)

**DOI:** 10.1038/s41598-021-87863-y

**Published:** 2021-04-15

**Authors:** Ha-Young Rhim, Sae-Yeon Won, Sepide Kashefiolasl, Nina Brawanski, Elke Hattingen, Joachim Berkefeld, Volker Seifert, Juergen Konczalla

**Affiliations:** 1 Department of Neurosurgery, University Hospital, Goethe University, Frankfurt am Main, Germany; 2 Institute of Neuroradiology, University Hospital, Goethe University, Frankfurt am Main, Germany

**Keywords:** Health care, Medical research, Neurology

## Abstract

Patient care in a neurointensive care unit (neuro-ICU) is challenging. Multidrug-resistant organisms (MDROs) are increasingly common in the routine clinical practice. We evaluated the impact of infection with MDROs on outcomes in patients with subarachnoid hemorrhage (SAH). A single-center retrospective analysis of SAH cases involving patients treated in the neuro-ICU was performed. The outcome was assessed 6 months after SAH using the modified Rankin Scale [mRS, favorable (0–2) and unfavorable (3–6)]. Data were compared by matched-pair analysis. Patient characteristics were well matched in the MDRO (n = 61) and control (n = 61) groups. In this center, one nurse was assigned to a two-bed room. If a MDRO was detected, the patient was isolated, and the nurse was assigned to the patient infected with the MDRO. In the MDRO group, 29 patients (48%) had a favorable outcome, while 25 patients (41%) in the control group had a favorable outcome; the difference was not significant (p > 0.05). Independent prognostic factors for unfavorable outcomes were worse status at admission (OR = 3.1), concomitant intracerebral hematoma (ICH) (OR = 3.7), and delayed cerebral ischemia (DCI) (OR = 6.8). Infection with MRDOs did not have a negative impact on the outcome in SAH patients. Slightly better outcomes were observed in SAH patients infected with MDROs, suggesting the benefit of individual care.

## Introduction

Subarachnoid hemorrhage (SAH) is a severe brain injury associated with high morbidity and mortality. It requires immediate hospital admission following endovascular or microsurgical treatment^1^. Previously, several factors like admission status according to the World Federation of Neurosurgical Societies scale (WFNS), the development of delayed cerebral ischemia (DCI), cerebral vasospasm (CVS), location of the aneurysmal bleeding, the presence of intracerebral hematoma (ICH), and the occurrence of an inflammatory reaction were identified as independent predictors for functional outcome in patients with SAH^2–5^.

After treatment of aneurysm, patients receive intensive care up to 40 days in a neuro-intensive care unit (neuro-ICU)^6,7^. The delivery of care in a neuro-ICU is associated with numerous complications and relatively high mortality^6–10^. Infections, particularly with multidrug-resistant organisms (MDROs), are serious complications associated with extended hospital stays, increased costs and are considered predictive of a worse outcome^11–14^. Furthermore, growing antibiotic resistance makes MDROs an increasing challenge in the routine clinical practice^11,12,15,16^. However, the impact of infection with MDROs on the outcome in patients with SAH has been elusive. Thus, we assessed the impact of nosocomial infection with MDROs and subsequent isolation on the outcome in SAH patients in this study.

## Results

### Patient characteristics

During the study period, MDROs were identified in 61 of 1237 SAH patients (4.9%). The mean age was 57 years (range 15–90). Forty-five (73.7%) of these patients were female, 17 (27.9%) were smokers, and 34 (55.7%) had a poor status at admission according to the WFNS (4 and 5). A total of 51 patients (84%) had a Fisher 3 or 4 bleeding type. Brain damage was defined either as concomitant ICH or DCI (territorial, border and multiple infarction syndrome). Concomitant ICH was found in 19 (31.1%) patients, and DCI was found in 35 (57.4%) patients. Twelve (19.7%) patients had both ICH and DCI. These characteristics are shown in Table 1.

**Table 1:** Patient characteristics.

	MDRO group (n = 61)	Matched control group (n = 61)	P*
Mean age, years	57	57	NS
Female sex	45 (73.8%)	42 (68.9%)	NS
**Admission status according to WFNS**			
Good (WFNS I–III) Poor (WFNS IV–V)	27 (44.3%) 34 (55.7%)	28 (45.9%) 33 (54.1%)	NS
**Total number of aneurysms**			
1 2 3 4 5	38 (62.3%) 16 (26.2%) 4 (6.6%) 2 (3.3%) 1 (1.6%)	45 (73.8%) 12 (19.7%) 4(6.6%) 0 (0.0%) 0 (0.0%)	NS
**Size of aneurysm**			
0–5 mm 6–10 mm 11–15 mm 16–20 mm	26 (42.6%) 21 (34.4%) 6 (9.8%) 2 (3.3%)	24 (39.3%) 26 (42.6%) 5 (8.2%) 2 (3.3%)	NS
Intracerebral hemorrhage	19 (31.1%)	15 (24.6%)	NS
Fisher grade 3/4 blood pattern	51 (83.6%)	56 (91.8%)	NS
**Cerebral vasospasm**			
< 33% 33–66% > 66%	23 (37.7%) 13 (21.3%) 25 (41.0%)	19 (31.2%) 10 (16.4%) 32 (52.5%)	NS
Treatment of CVS with nimodipine	11 (18.0%)	8 (13.1%)	NS
Early hydrocephalus	48 (78.7%)	54 (88.5%)	NS
**Delayed infarctions**			
Territorial Boarder Multiple infarction syndrome	35 (57.4%) 23 (37.7%) 4 (6.6%) 8 (13.1%)	44 (72.1%) 27 (44.3%) 7 (11.5%) 10 (16.4%)	NS
Smoking	17 (27.9%)	21 (34.3%)	NS
Shunt dependency after 6 months	20 (32.8%)	19 (31.2%)	NS
**Outcome after 6 months according to the modified Rankin scale**			
0–2 favorable 3–6 unfavorable	29 (47.5%) 32 (52.5%)	25 (41.0%) 36 (59.0%)	NS

*WFNS* World Federation of Neurological Surgeons, *CVS* cerebral vasospasm, *MDRO* multidrug resistant organism; categorial variables were analyzed using the Fisher exact test and unpaired t-test with Welch correlation (*) for parametric values. Probability values < 0.05 were considered statistically significant. NS, not significant (P > 0,05).

### Distribution of the MDROs

**Figure 1:**
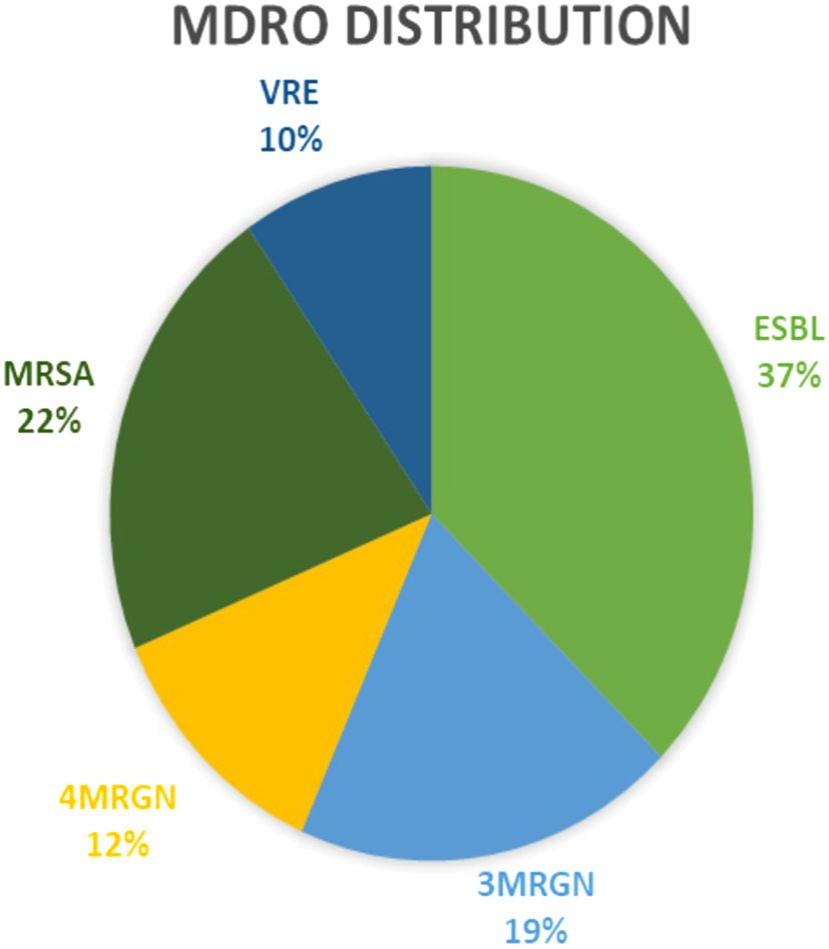
MDRO distribution. *MDRO* multi drug resistant organism, distribution in the subgroup. *ESBL* extended-spectrum beta-lactamase producing bacteria (*E.coli, K. pneumoniae, K. oxytoca*), *MRSA* methicillin-resistant *Staphylococcus aureus*, *3 MDRGN* multidrug resistant Gram-negative bacteria (*E. coli, K. pneumoniae, P. aeruginosa*), *4 MDRGN* multidrug resistant Gram-negative bacteria (*A. baumanii, K. pneumoniae, P. aeruginosa, E. aerogenes*), *VRE* vancomycin-resistant *Enterococcus*. 4 patients with multiple infections (2 patients 3 MDRGN + VRE, 1 patient MRSA + ESBL, 1 patient VRE + 3MDRGN) resulting in 83 positive detections in total.

The following MDROs were identified: Extended-spectrum β-lactamase (ESBL)-producing bacteria (37.3%) (*Escherichia coli*, *Klebsiella pneumoniae*, *K. oxytoca*), methicillin-resistant *Staphylococcus aureus* (MRSA) (21.7%), 3 Multidrug-resistant gram negative rods (3 MDRGNs) (19.2%) (*E. coli*, *K. pneumoniae*, *Pseudomonas aeruginosa*), 4 Multidrug-resistant gram negative rods (4 MDRGNs) (12.0%) (*Acinetobacter baumannii*, *K. pneumoniae*, *P. aeruginosa*, *K. aerogenes*) and Vancomycin-resistant enterococci (VRE) (9.6%). A total of 4 patients had multiple infections (2 patients had 3 MDRGN + VRE, 1 patient had MRSA + ESBL, 1 patient had VRE + 3 MDRGN). Overall, 83 infections were identified (Fig. 1).

### Matched-pair analysis

Sixty-one patients in our database were in the control group (1:1 matched-pair analysis). In addition to the selected parameters (patient age, admission status, early hydrocephalus, Fisher score, and treatment procedure), the groups were well matched regarding other potential factors associated with the outcome (additional ICH, smoking status, number and size of aneurysms, and shunt dependency after six months). The clinical course showed similar rates of CVS and DCI in the two groups (see Table 1). The distribution of parameters was very similar between the MDRO subgroup and all patients enrolled in the study.

### Clinical outcome

After six months, 29 patients in the MDROs (48%) and 25 patients in the matched control group (41%) had a favorable outcome; the difference was not significant (p > 0.05). Shunt dependency after six months was identified in 20 (32.8%) patients in the MDRO group and in 19 (31.1%) patients in the control group. In total, six (9.8%) patients in the MDRO subgroup died during hospitalization. Of them, two (3.3%) patients died due to severe sepsis associated with infection with MDROs (1 patient with MRSA, 1 patient with 4 MDRGN). The other 4 patients died due to noninfectious causes.

### Prognostic factors in the MDRO subgroup

A total of 32 (52%) patients in the MDRO group had an unfavorable outcome (Table 2). Significant predictors of an unfavorable outcome were a worse status at admission (OR = 3.1), ICH (OR = 3.7) and DCI (OR = 6.8).

**Table 2:** Predictors of an unfavorable outcome in the MDRO subgroup.

	Favorable outcome (n = 29)	Unfavorable outcome (n = 32)	OR (CI)	P*
Mean age, years	58	56		NS
Female sex	20 (69.0%)	25 (78.1%)		NS
**Admission status according to WFNS**				
Good (I–III) Poor (IV–V)	17 (58.6%) 12 (41.4%)	10 (32.3%) 22 (68.8%)	3.12 (1.09–8.92)	**0.041**
Intracerebral hemorrhage	5 (17.2%)	14 (43.8%)	3.73 (1.14–12.27)	**0.031**
Fisher grade 3/4 blood pattern	23 (79.3%)	28 (87.5%)		NS
Severe cerebral vasospasm	11 (37.9%)	14 (43.8%)		NS
Early hydrocephalus	23 (79.3%)	25 (78.1%)		NS
Delayed cerebral infarctions	10 (34.5%)	25 (78.1%)	6.79 (2.18–21.12)	** < 0.001**
Smoking	7 (24.1%)	10 (31.3%)		NS
Anticoagulant therapy	3 (10.3%)	2 (6.3%)		NS
Shunt dependency after 6 months	7 (24.1%)	13 (40.6%)		NS

Outcome assed according to the modified Rankin Scale (mRS, favorable 0–2, unfavorable 3–6).

*OR* odds ratio, *CI* confidence interval, *MDRO* multidrug resistant organism, *WFNS* World Federation of Neurological Surgeons.

## Discussion

During the study period, MDROs were identified in 4.9% of patients with SAH, which is comparable to other German and French data^17,18^.

Usually, patients with SAH require prolonged stays in the neuro-ICU, which is associated with an increased risk for colonization or infection with multidrug-resistant bacteria^19–21^. Infections with multidrug-resistant pathogens necessitate antibiotic treatments, compliance with hygiene standards (such as contact isolation), and increased attention from medical staff^16,20,22^.

In the current study, we examined the outcome in patients with SAH and infection or colonization with multidrug-resistant pathogens who were isolated in the neuro-ICU and compared them with outcome in SAH patients not infected or colonized with MDROs.

The treatment of patients with SAH is particularly challenging and requires an awareness of all potential neurological and medical complications^23–25^. Hospital isolation seems to be associated with adverse effects, which may result in worse hospital outcome^14,19^. However, there have been no studies involving SAH patients admitted to a neuro-ICU and isolated due to MDRO colonization and/or infection. Therefore, we reviewed our prospectively collected data to evaluate potential differences between these patients and patients without infections who were not isolated. Sixty-one patients were included, and a matched-pair analysis was performed to exclude confounding effects. Except for isolation and, if necessary, antibiotic therapy, both groups received the same treatment. Recently, Tran et al. reported that hospital isolation was associated with an increased length of stay (LOS)^19,20^. As mentioned before, the LOS is closely linked to adverse events and nonmedical complications caused by clinical staff^19–21^. These complications are believed to be the result of understaffing and increased nurse workload, among other things^7,10^. However, our findings did not show significant differences between the subgroup of patients infected or colonized with MDROs and the matched control group. The average length of stay was 57 days in the MDRO group (range 15–90 days), and it was also 57 days in the control group (range 27–82 days), including treatment in neurological rehabilitation centers. Overall, our results show that neither colonization or infection with multidrug-resistant agents nor the subsequent isolation leads to a worse outcome. This result is surprising. Our trial involved several factors that might influence the clinical outcome, such as a stay in the neuro-ICU, colonization or infection with MDROs and contact isolation, which led us to expect significant or at least a tendency towards a significant difference between the MRDO and control groups. Interestingly, a slight tendency towards a better outcome in the MDRO group was observed, indicating that personalized attention (1:1 care) could improve the outcome.

Our results suggest that infection or colonization with MDROs does not necessarily result in a worse outcome when there is good patient and human resource management (1 nurse per room), high quality staff, and compliance with health standards.

Deptula et al. compared virulence factors in multidrug-resistant (MDR) and multidrug-sensitive (MDS) *P. aeruginosa* (PA) strains in an in vitro study^26^. The growth rate and the production of extracellular material capable of binding to Congo red were significantly lower in the MDR strains than in the MDS strains. The same results were found regarding lipolytic, elastase, LasA protease, and phospholipase C activity and the quantity of pyocianin. The MDR PA strains were significantly less virulent than the MDS PA strains. This result was supported by an in vivo model investigated by Zorrilla-Gomez et al.^27^. As part of a peritonitis/sepsis model, one group of mice was inoculated with MDR PA strains, and the other group was inoculated with MDS PA strains. The probability of mortality at 48 h was significantly higher in those inoculated with the MDS strains (MDS 75.0% versus MDR 7.5%). Furthermore, the bacterial concentrations in the peritoneal fluid were higher in the mice inoculated with MDS strains, indicating a relatively higher growth rate of those strains. These experimental studies may help explain why infection with MDROs was not a significant factor predictive of a worse outcome^26–29^.

Indeed, several attributes were not considered in our evaluation: comorbidities, the social context of the patients, the patients’ mental status after ictus, patient adherence to medical treatment, the quality of the posthospital rehabilitation facilities, and, particularly, the differences in pathogenicity of particular MDROs. We did not differentiate among the multidrug-resistant organisms but rather classified them into five categories (MRSA, VRE, ESBL-producing, 3 MDRGN, and 4 MDRGN) and did not consider the possible differences in pathogenicity. Although infection with *A. baumannii* rarely occurred at our center during the observation period (n = 3 in 12 years), it is presumed to be associated with increased mortality and a worse clinical outcome^30–32^. Nonetheless, in our center, the clinical significance of *A. baumannii* infection remains limited due to its rarity. Infection with *K. pneumoniae* also appears to be associated with higher mortality^8,9,13^. In contrast, infection with MRSA did not appear to have an impact on the clinical outcome^33^.

The study has several limitations. It was a retrospective, single-center statistical analysis with a relatively small study population. Although the data were collected prospectively, the retrospective design has inherent limitations, such as missing or unavailable data if the data were not initially documented in the medical records. In this analysis, we did not consider the severity of the infection or the length of isolation. A larger population of patients must be evaluated to validate the results of our analysis and enable firm conclusions to be drawn. Therefore, we suggest the performance of a larger multicenter trial with more participants to evaluate the effect of single-agent pathogenicity on the clinical outcome.

We highly recommend observing isolated MDRO patients in the neuro-ICU. However, the focus should be on preventing contamination/infection and applying a tailored antibiotic therapy as soon as possible to minimize the risk of the spread of the infection and the development of a more extended spectrum of resistance.

Despite the mentioned weaknesses, we believe that our results are important for the management of SAH patients in a neuro-ICU.

## Materials and methods

A total of 1237 patients with SAH were treated in our institute between 2003 and 2015 and were prospectively included in our patient registry (IBM SPSS Statistics, version 22, Armonk, NY, USA). Patient characteristics, treatment type, radiological features, initial results, clinical course, and outcomes during follow-up were prospectively collected^34^.

Patient data were anonymized; therefore, consent for publication was not required. The patient registry was used in a retrospective study. The inclusion criteria were SAH patients presenting with an additional infection with at least one MDRO. Variables were compared by matched-pair analysis between the patients with and without MDRO infections. The aim of the study was (1) to compare the clinical outcomes between SAH patients with or without MDRO infections and (2) to evaluate predictors of favorable/unfavorable outcomes in patients with MDRO infections in a subgroup analysis.

### Patient management

The diagnosis of SAH was established at admission by computed tomography (CT) scan or lumbar puncture. The amount of subarachnoid blood was assessed according to the modified Fisher score^35^. A rupture of a cerebral aneurysm as the origin of hemorrhage was confirmed by 4-vessel 3D angiography. Aneurysms were regularly treated by coiling or clipping within 24 h based on interdisciplinary consensus. In the case of early hydrocephalus, treatment included the placement of an external ventricular drain in loco typico.

All patients were treated in the neuro-ICU. A central venous line was placed for infusions, and an arterial line was placed for sequential blood gas analyses and blood pressure management.

During hospitalization in the neuro-ICU, the treatment included screening for CVS by regular clinical examinations and transcranial Doppler ultrasound. In cases of potential CVS, additional imaging was performed (CT angiography, magnetic resonance angiography or digital subtraction angiography). Severe CVS was defined as arterial vessel narrowing greater than 66% on radiological imaging. Patients with confirmed CVS were treated with hypertension (cerebral perfusion pressure over 90 mmHg) and, in certain cases, with intraarterial nimodipine.

### ICU management

Our neuro-ICU has only rooms with two beds. Between 2003 and 2015, the neuro-ICU was enlarged from 14 to 16 mechanical ventilation stations (from 7 to 8 rooms). If an MDRO was detected, the patient and his roommate were separated and isolated. After testing negative for MDROs, the roommate was no longer isolated. The work was organized in a three-shift system. Every nurse managed one room with two patients. When patients with MDRO infections were isolated, the number of nurses was not adjusted because the nurses needed more time for appropriate hygiene practices and changes of protective gear; therefore, each nurse managed one room with one isolated patient. Patients’ relatives and visitors also had to use protective gear.

Treatment in the neuro-ICU includes daily clinical assessments, arterial blood gas analyses, invasive blood pressure (IBP) measurement with either catecholamine or vasopressin, sedation, antiedema treatment and invasive ventilation with weaning as the clinical course progresses. Moreover, bedsore prevention measures, frequent nutritional support and blood tests require significant effort on the part of the medical staff. Considering these challenging circumstances, the ICU management of patients with SAH is associated with a prolonged hospital stay, high costs, and adverse effects that may result in worse outcomes.

### Multidrug-resistant organisms

Smear tests were routinely performed at admission and twice a week thereafter. Patients who tested positive for MDROs were strictly isolated from patients who tested negative according to the hygiene regulations of the hospital.

We considered vancomycin-resistant enterococci (VRE), methicillin-resistant *Staphylococcus aureus* (MRSA), extended-spectrum β-lactamase (ESBL)-producing bacteria and multidrug-resistant gram-negative (MDRGN) bacilli, which were divided into 3 MDRGN and 4 MDRGN bacteria, to be multidrug-resistant organisms. A positive test in patient samples was the basis for the diagnosis of infection with an MDRO. Therefore, swab tests of the nasopharynx, wounds, inguinal region, and perianal and vaginal regions were performed routinely at least twice a week. If necessary, microbiological examinations of tracheal secretions, urine and central venous line tips were performed. When bacteremia was suspected, blood swab tests and blood cultures were performed. Patient samples were sent to the in-hospital laboratory immediately. Patients with positive test results were treated according to the result of antibiotic sensitivity testing (AST), and the treatment was continued until a negative test result was obtained. For identification of MDROs and performance of AST, VITEK2 (bioMérieux, Marcy l'Etoile, France) was used. AST was assessed in accordance with the breakpoint tables of the European Committee on Antimicrobial Susceptibility Testing (EUCAST).

### Outcome assessment

The outcome was assessed according to the modified Rankin Scale (mRS) after six months. A mRS score of 0–2 was considered a favorable outcome, and a mRS score of 3–6 was considered an unfavorable outcome.

### Statistical analysis

For the statistical analysis, IBM SPSS Statistics was used (version 22, IBM Corp., Armonk, NY, USA). An unpaired *t*-test was used for parametric analyses. Categorical variables were analyzed in contingency tables using χ^2^ tests. A p-value < 0.05 was considered statistically significant.

For the matched-pair analysis, the statistical computing program R (version 3.0.3; The R Foundation for Statistical Computing, https://www.r-project.org/) was used.

After identifying MDRO patients, we performed multivariate analyses and propensity score matching with balance optimization. Patients without MDRO infections were selected (using R) as a control group. The following factors that could affect the outcome were selected for matching: patient age, admission status, early hydrocephalus, modified Fisher score and treatment procedure (coiling/clipping/combined).

### Ethical approval

The study was approved the ethics committee of the University Hospital, Goethe University, Frankfurt am Main, Germany (Ethik-Kommission des Fachbereichs Medizin Universitätsklinikum der Goethe-Universität) and the informed consent was waived by the ethics committee of the University Hospital, Goethe University, Frankfurt am Main, Germany (Ethik-Kommission des Fachbereichs Medizin Universitätsklinikum der Goethe-Universität). All methods were carried out in accordance with relevant guidelines and regulations.

## Abbreviations

AST: Antibiotic sensitivity testing
CT: Computer tomography
CVS: Cerebral vasospasm
DCI: Delayed cerebral ischemia
ESBL: Extended-spectrum β-lactamase
IBP: Invasive (intra-arterial) blood pressure
ICH: Intracerebral hemorrhage
ICU: Intensive care unit
LOS: Length of stay
MDR: Multidrug-resistant
MDS: Multidrug-sensitive
MDRGN: Multidrug-resistant gram negative rods
MDRO: Multidrug-resistant organism
mRS: Modified Rankin Scale
MRSA: Methicillin-resistant staphylococcus aureus
SAH: Subarachnoid hemorrhage
VRE: Vancomycin-resistant enterococci
WFNS: World Federation of Neurological Surgeons
